# Optimisation of Stingless Bee Honey Nanoemulsions Using Response Surface Methodology

**DOI:** 10.3390/foods10092133

**Published:** 2021-09-09

**Authors:** Azri Shahir Rozman, Norhashila Hashim, Bernard Maringgal, Khalina Abdan

**Affiliations:** 1 Department of Biological and Agricultural Engineering, Faculty of Engineering, Universiti Putra Malaysia, Serdang 43400, Selangor, Malaysia; GS60694@student.upm.edu.my (A.S.R.); khalina@upm.edu.my (K.A.); 2 SMART Farming Technology Research Centre (SFTRC), Faculty of Engineering, Universiti Putra Malaysia, Serdang 43400, Selangor, Malaysia; 3 Department of Agriculture Malaysia, Putrajaya 62624, Malaysia; bernardmaringgal@yahoo.com

**Keywords:** stingless bee honey, nanoemulsions, high-pressure homogeniser, response surface methodology, oleic acid

## Abstract

Nanoemulsions (NEs) have been used in a wide range of products, such as those produced by the food, cosmetics, and pharmaceutical industries, due to their stability and long shelf life. In the present study, stingless bee honey (SBH) NEs were formulated using SBH, oleic acid, tween 80, glycerol, and double-distilled water. SBH NEs were prepared using a high-pressure homogeniser and were characterised by observing their stability and droplet size. Fourier Transform-Infrared (FTIR) analysis was used to observe the functional groups of the SBH NEs after being subjected to high-pressure homogenisation. Transmission Electron Microscopy (TEM) images were then used to confirm the particle size of the SBH NEs and to investigate their morphology. The effects of the independent variables (percentage of oleic acid, storage time, and storage temperature) on the response variables (particle size and polydispersity index) were investigated using the response surface methodology, along with a three-level factorial design. The results showed that the models developed via the response surface methodology were reliable, with a coefficient of determination (*R*^2^) of more than 0.90. The experimental validation indicated an error of less than 10% in the actual results compared to the predicted results. The FTIR analysis showed that SBH NEs have the same functional group as SBH. Observation through TEM indicated that the SBH NEs had a similar particle size, which was between 10 and 100 nm. Thus, this study shows that SBH NEs can be developed using a high-pressure homogeniser, which indicates a new direction for SBH by-products.

## 1. Introduction

Stingless bees are highly eusocial insects and can be found in tropical and subtropical regions. They belong to the Hymenoptera order, under the *Apidae* family and *Meliponini* sub-family. Similar to *Apis* bees, the stingless bees also produce and store honey in their hive [1]. Studies have shown that stingless bee honey (SBH) contains antimicrobial activity against bacteria, yeast, and fungal strains [2]. Further, SBH is considered to be a premium product with a market value that may reach up to $100/kg, which is approximately twice the price of the honey of the *Apis* bee, which ranges from $20 to $40/kg [3]. In Malaysia, the SBH industry is expanding due to its potential and benefits. According to the Malaysian Department of Agriculture [4], the total farming area of stingless bees was reported to be about 1606.6 ha in 2017 and this figure was expected to increase yearly. 

The composition of SBH consists of a mixture of carbohydrates, glucose, fructose, amino acids, organic acids, minerals, aromatic substances, vitamins, pigments, beeswax, and pollen, all of which affects its colour, smell, and taste. In addition, water is one of the main compositional elements of SBH and is considered a highly important feature that can determine the viscosity, specific weight, maturity, flavour, and crystallisation properties [5]. Abu Bakar et al. [6] reported that SBH possesses medicinal and therapeutic value. SBH has been proven to have positive effects as an antimicrobial and anticancer agent, as well as improving hypertension and lipid profiles, and demonstrating antidiabetics effects which are demonstrably better than other types of honey [7]. The physicochemical properties of SBH vary according to the type of flora, climate, and geographical features of the region in which the bees collect the raw materials for consumption [2,8].

Awareness of nanoemulsions (NEs) has increased exponentially over the past decades due to their uniqueness in terms of physicochemical properties and the potential application as a delivery system for bioactive molecules [9]. Industries such as cosmetics, pharmaceuticals, food, etc., find NEs an attractive system due to their low amount of surfactant, higher stability, low viscosity, good appearance, and versatility in their formulation, as well as characteristics that are less toxic or irritating [10]. An NE is a heterogeneous system that contains two immiscible liquids in which one phase is dispersed into nanoparticles with a droplet radius ranging from 10 to 100 nm [11]. Further, NEs have several unique characteristics compared to conventional emulsions, such as a longer shelf life, improved bioavailability, and greater physical stability [12]. Salvia-Trujillo et al. [13] stated that their study of antimicrobial NEs has opened a novel view in producing an edible coating for assurance of the safety and quality of fresh/fresh-cut fruits. 

There are two methods for producing NEs which involve either low energy or high energy. The low-energy method is less expensive and does not require sophisticated equipment. However, the low-energy method is only efficient at producing NEs limited to a certain combination of oils and emulsifiers. As for the high-energy method, it involves a mechanical component, forcing oil droplets to break up into a finer size that can disperse into water [14]. In the pharmaceutical field, high-pressure homogenisation (HPH) is widely used due to its scalability as a high-energy emulsification method [15]. Originally, HPH was used to stabilise pasteurised milk by disrupting fat globules, thus decreasing the creaming rate. Nowadays, HPH is used for emulsifying low- to moderate-viscosity fluids [16]. HPH has been used to provide good texture characteristics and higher stability in food production emulsions [17]. A study by Hidajat et al. [18] showed that limonene NEs produced using HPH were highly stable for over 28 days. The limonene NEs’ stability and particle size were affected by the number of passes and pressure used during the homogenisation process. A study by Akbas et al. [19] also has proven that capsaicin-loaded NEs produced using HPH have better antimicrobial activity than those produced using the ultrasonication method, despite both methods showing good stability for 28 days and particle size within desired range.

The response surface methodology (RSM) is a statistical procedure usually used for optimisation studies, especially in food processing, as it provides an extension of the parameters space studied and simultaneously reduces the number of experiments [20]. A study by Mehmood [21] on canola oil NEs produced using HPH that was optimised using RSM showed the model and equation developed were within the expected results. The author stated that the optimum conditions for producing the NEs were a homogenization pressure of 135 MPa and a concentration of 6.18% oil, 6.39% surfactant and 1% vitamin E acetate. Another study using RSM in optimising NEs, by Samiun et al. [22], also proved that RSM is an efficient tool to produce desired characteristics in NEs. The author found that to produce optimum aripiprazole NEs using HPH, the parameters needed were overhead stirring for 15 min, a 4400 rpm shear rate, and 11 cycles of HPH. Further, a study by Izadiyan et al. [23] on sorafenib NEs for cancer treatment found that to produce optimum NEs, the parameters needed were overhead stirring for 120 min, a 4000 rpm shear rate, and 16 cycles of HPH. These studies indicate the capability of RSM in optimising the processing of food.

The study of SBH in various fields is increasing globally as people are starting to become aware of its benefits. SBH has been used as an edible coating for fresh produce [24], as well as in the clinical field by Rashid et al. [25] and Yazan et al. [26], among others, due to its antimicrobial and antioxidant properties. However, studies on the application of NE technology to SBH, which could offer better efficiency and many other benefits, are still limited in number. The food, beverage, cosmetic, personal care, and pharmaceutical industries have been using emulsions and NE systems widely in their products. In addition, the demand from consumers for commercial products to use healthy and safe ingredients is increasing [11]. This proves that health and safety concerns are very important. Organic or natural substances, especially SBH, need to be explored as the base of products. Furthermore, in terms of SBH in the form of NEs, information is very scarce, and of that which does exist, only data regarding typical honey emulsions are available. Hence, it is important to investigate the characteristics and morphology of SBH in NE form for a better understanding of this substance and its future use.

Thus, the main objective of this study is to investigate the formulation of SBH NEs using HPH. The specific objectives are: (1) To identify the optimum method for SBH NE formulation by means of the response surface methodology (RSM); (2) To analyse the characterisation of SBH and SBH NEs using Fourier-Transform Infrared Spectroscopy (FTIR) and Transmission Electron Microscopy (TEM).

## 2. Materials and Methods

### 2.1. Formulation and Preparation of SBH NEs

SBH was purchased from a local bee farmer and stored in an airtight container to avoid any moisture disturbance. The container was kept at room temperature before proceeding with the experiments. SBH NEs were formulated using SBH as a base ingredient, along with glycerol, tween 80, oleic acid, and distilled water. Glycerol is a short-chain alcohol that is colourless, odourless, and non-toxic; it acts as a stabiliser for NEs. Tween 80 is a surfactant used when producing NEs. According to Chong et al. [27], a surfactant helps the dispersion of all NE components by reducing interfacial tension. Oleic acid is a pale yellow liquid with a faint odour and acts as the oil component and co-surfactant in producing NEs. Three different formulations were used to produce SBH NEs, labelled A1, A2, and A3. They varied in their oleic acid and distilled water content, but the percentages of glycerol, surfactant (Tween 80), and SBH were fixed. Formulations A1, A2, and A3 were prepared with 1% (*w*/*w*), 3% (*w*/*w*), and 5% (*w*/*w*) of oleic acid, respectively. Table 1 shows the percentage of materials used to produce formulations A1, A2, and A3. All materials were mixed together in a beaker and subjected to a magnetic stirrer for 2 h at room temperature to produce a pre-emulsion. Then, SBH NEs were produced by subjecting the pre-emulsions to HPH (Avestin Canasa C50) for 5 cycles at 100 MPa at room temperature. Each formulation was then kept at three different temperatures—at room temperature, at 4 °C, and at 40 °C—for three weeks.

### 2.2. Characterisation of SBH NEs

#### 2.2.1. Particle Size and Polydispersity Index Analysis

The particle size and polydispersity index (PDI) of the SBH NEs was analysed using a particle size analyser (Nanoplus Zeta/Nanoparticle Analyser, Kyoto, Japan) at room temperature. The sample was initially diluted with distilled water at a ratio of 1:10 directly in a disposable cuvette. Then, measurement of the sample was carried out automatically by placing the cuvette in the particle size analyser. The particle size of the SBH NEs was recorded in nm; PDI is a dimensionless value.

#### 2.2.2. Stability

The stability of SBH NEs was determined by recording the particle size and capturing the appearance on days 0, 7, and 14 under storage at room temperature, 4 °C, and 40 °C. The appearance of the SBH NEs was observed to ensure it could maintain a uniform phase and colour. The best SBH NE formulation was determined using the response surface methodology (RSM).

#### 2.2.3. Fourier-Transform Infrared Spectroscopy (FTIR)

The detection of possible changes in the existing functional groups in the SBH and SBH NEs was performed with the aid of FTIR spectroscopic measurements using a Perkin Elmer Spectrum 100 (PerkinElmer, Waltham, MA, USA), in the wave number range of 650 to 4000 cm^−1^. The spectral resolution was 2 cm^−1^ and 16 scans were used. As a reference for the honey and SBH NE sample, the absorption bands corresponding to chemical compounds were identified in the respective samples and the appropriate spectra-processing procedures were followed. First, a spectrum of air was collected under the same conditions as the spectra of the investigated SBH NEs. Then, the absorbance spectrum of the air was automatically subtracted from the spectra of the samples to obtain only the FTIR spectra of the SBH NEs. Each measurement was performed in triplicate. In all the figures, the average of the triplicate measurements of the obtained spectra for each sample is presented.

#### 2.2.4. Transmission Electron Microscopy (TEM)

The morphology and particle size of a selected formulation were analysed using a Field Emission Electron Microscope ( JEM-2100F, Jeol, Japan) (JEM-2100 F) with a working voltage of 200 kV. The NEs were diluted with deionised water by 20 times and applied onto a 300-mesh copper grid for 1 min. Then, the grid was kept inverted and a drop of tungstic acid (PTA 1%) was applied to the grid for 10 s. Excess PTA was removed using filter paper.

### 2.3. Response Surface Methodology (RSM)

A three-level factorial design—specifically, a statistical experiment with 3 factors, 3 levels, and 27 runs—was selected for the optimisation study. The optimisation of SBH NEs was performed using Design Expert 11 software. An analysis of variance (ANOVA) between the mean of the samples was conducted with a confidence level of 95%. Any significant differences among the samples were determined using Fisher’s test, and all the data were reported as mean ± standard deviation. The optimum formulation of SBH NEs was obtained by analysing the response surfaces and solving the regression equation. The fitted polynomial equations were expressed in three-dimensional response surface plots to explain the interaction effects of independent variables on the dependant variables. The regression equations can be expressed as:

Y = β + βA + βB + βC + βAB + βAC + βBC + βA^2^ + βB^2^ + βC^2^ (1)

where Y demonstrates the predicted response; β is the intercept; βA, βB, βC are linear coefficients; βAB, βAC, βBC are the interactions coefficients; and βA^2^, βB^2^, βC^2^ are the squared coefficients. 

## 3. Results and Discussion

### 3.1. Identification of Optimum SBH NE Formation

#### 3.1.1. The Physical Observation of SBH NEs

**Figure 1:**
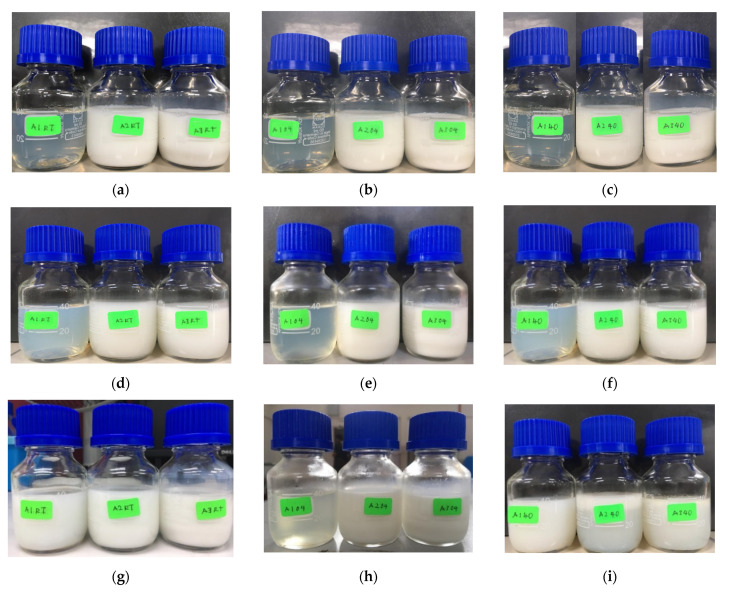
The changes in physical appearance of SBH NEs. From left to right, the samples are denoted as A1, A2, and A3, kept for 0 days’ storage at room temperature (**a**), 4 °C (**b**) and 40 °C (**c**); 7 days’ storage at room temperature (**d**), 4 °C (**e**), and 40 °C (**f**); and 14 days’ storage at room temperature (**g**), 4 °C (**h**), and 40 °C (**i**).

The changes in the physical appearance of the SBH NEs are shown in Figure 1. Referring to Figure 1, the initial appearance of formulation A1 was translucent, and formulations A2 and A3 were milky. The appearance of the formulations may have been be affected by the percentage of oleic acid, of which formulation A1 had the lowest percentage, at 1% (*w*/*w*). Subsequently, during the seven days of storage, the appearance of formulation A1 became cloudy at temperatures of 4 °C and 40 °C, whereas at room temperature the appearance remained constant. On the other hand, the appearance of formulations A2 and A3 remained milky at all three temperatures throughout the seven days of storage. 

The observation on day 14 showed that formulation A1 became milky at room temperature and 40 °C but remained translucent at a temperature of 4 °C. Formulation A2 and A3 did not show any changes during storage at 14 days. The milky appearance of formulation A2 and A3 may be due to the high concentration of oleic acid. According to Ricaurte et al. [28], even though oleic acid has emulsifying attributes, it is also highly hydrophobic, which tends to impede its surfactant action. Akbas et al. [19] also found that their NEs went through phase changes and produced clumps. This was caused by the reduction of droplet size and turbidity value, although the droplet size was still in the nanoscale range. In addition, a study by Feng et al. [29] also showed a milky appearance for some of their pesticide NEs during 14 days of storage.

#### 3.1.2. Stability Analysis and Optimisation for Best SBH NE Formulation

**Figure 2:**
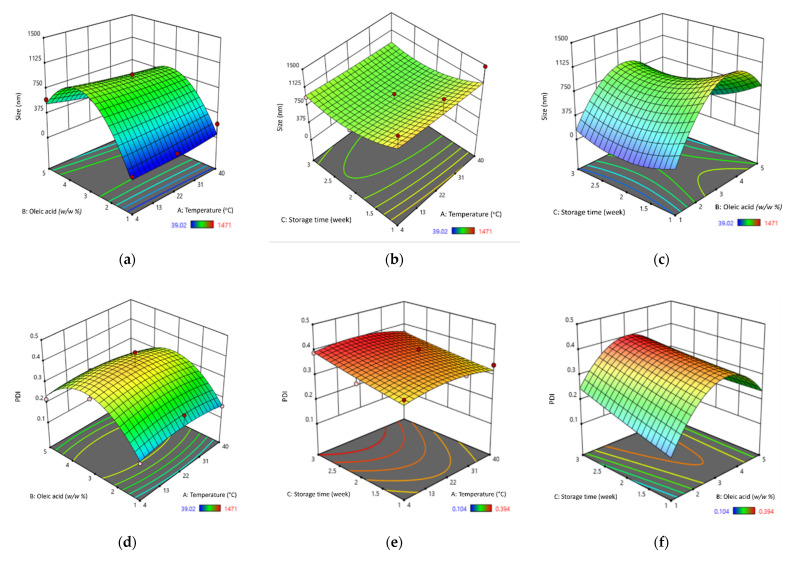
Response surface plot of the particle size of SBH NEs in response to the combined effect of temperature and oleic acid (**a**), temperature and storage time (**b**), and storage time and oleic acid (**c**); Response surface plot of the PDI of SBH NEs due to the combined effect of temperature and oleic acid (**d**), temperature and storage time (**e**), and storage time and oleic acid (**f**). A red dot represents points above the predicted value and a pink dot represents points below the predicted value.

A stability analysis was performed using RSM to acquire the best formulation for an SBH NE. A three-level factorial design experiment was chosen, for which the factors were temperature (A), oleic acid percentage (B), and storage time (C). In addition, the desired target characteristics for the model were a particle size between 10 and 100 nm and a PDI value between 0.08 and 0.70. Table 2 represents the factors with levels which are low (−1), middle (0), and high (1). The data were randomly arranged by the Design Expert 11 software and the response variables are presented in Table 3. Interactions between the characteristics of the SBH NEs and their independent variables of particle size and PDI are illustrated in Figure 2. The ANOVA results for the SBH NEs in terms of particle size and PDI are shown in Table 4. The statistical significance and adequacy of the models were determined using the Fisher *F*-test. The *F*-value (Fisher’s ratio value) and the *p*-value of the model indicated the significance of the overall model. The results showed that the *p*-values for both models were less than the *α*-value, indicating that all of the models were significant (Table 4). This was in agreement with a study by Yukuyama et al. [30] on olive oil NEs in which they reported the significance of their resultant quadratic polynomial model for the mean particle size of olive oil NEs. The authors reported that all the *p*-values of their model were less than 0.05, where the significant input factors of the particle size were pressure, surfactant, olive oil concentration, and interaction of surfactant with olive oil. 

Regarding the ANOVA of the two models in Table 4, temperature appeared to be insignificant, indicating that none of the temperatures used in this study had any significant effect on the particle size or PDI of the SBH NEs. Meanwhile, the other independent variables of both models—that is, percentage of oleic acid and storage time—were found to be significant, as their *p*-values were less than the *α*-value. This indicates that the percentage of oleic acid and storage time do indeed have a significant effect on the particle size and PDI of SBH NEs. In this work, oleic acid was used as the oil component in the NEs, at concentrations that varied among each of the SBH NE formulations. It was observed that the particle size and PDI were affected by the percentage of oleic acid used, due to its viscosity and the amount of energy supplied during homogenization, which is in agreement with the findings of Ricaurte et al. [28] in their study of high-oleic palm oil NEs. As for the effect of storage time on particle size and PDI, this was due to the particles in the NEs having a tendency to coalesce, which resulted in an increase in particle size and PDI value. The finding was in agreement with Chrastina et al. [31] in their study on plumbagin-loaded NEs, which indicated an increase in particle size and PDI over the course of three months of storage.

In order to determine which independent variables have a greater effect on the particle size and PDI, the *F*-values of all the variables were compared. Table 4 shows that the *F*-value of the percentage of oleic acid was almost double compared to the storage time (*F*-value_oleic acid_ = 35.24 > *F*-value_storage time_ = 18.68), which indicates that the percentage of oleic acid had more effect on the particle size. In contrast, the *F*-value for the percentage of oleic acid was higher than the storage time for PDI, at 22.20 compared to 15.11, indicating that storage time had a higher impact on PDI. The results of the ANOVA on particle size and PDI value of the SBH NEs were in agreement with Katsouli et al. [32] in their study of olive oil NEs, where a large *F*-value and low *p*-value of bioactive compound ratio and emulsifier ratio indicated a greater significant effect on the responsive variables, which were particle size, PDI, turbidity, and emulsion stability index. 

The interaction of storage time, percentage of oleic acid, and temperature can be seen clearly in Figure 2. The interaction between two independent variables, namely, storage time and percentage of oleic acid, had a positive influence on the particle size, whereas temperature had a negative influence on the particle size. Figure 2a shows the interaction of the percentage of oleic acid and different temperatures in affecting the particle size of SBH NEs, which indicates that temperature and percentage of oleic acid did not affect the size of the particles. In a study by Chen et al. [33], after the oleic acid percentage reached a critical value, the number of droplets would not increase. This was due to the coalescence of droplets and a decrease in emulsion homogeneity, which led to an uneven structure of the film. The graph shown in Figure 2b indicates that in our study, storage time and temperature did not significantly affect particle size. Similarly, in a study by Ruiz-Montañez et al. [34], the size of the droplet curve was not affected, as the formulation was kept at 4 °C and 20 °C. Further, the same result was obtained by Yukuyama et al. [30], who found that storage temperature during a stability test did not greatly affect the particle size, as the formulations were kept at 4 °C and 25 °C. Moreover, the interaction between the percentage of oleic acid and storage time affected the particle size in our study, as seen in Figure 2c, which shows that most of the particle sizes remained constant for each percentage of oleic acid. The smallest particle shown from the graph is the percentage of oleic acid at 1% (*w*/*w*). Ricaurte et al. [28] found that the percentage of oleic acid in a formulation may affect the particle size of the formulation, observing that as the concentration of oleic acid increased, the size of the particles also increased.

To quantify the homogeneity of the SBH NEs, PDI was used, as it measures the distribution of particle size. It was also important to determine the stability of the NEs [35]. Figure 2d shows the interaction of the percentage of oleic acid with temperature in affecting the PDI value. The results indicate that only the percentage of oleic acid contributes to PDI value. The PDI value for the interaction of storage time and temperature, as shown in Figure 2e, indicates that an increase in storage time could also increase the PDI. Figure 2f shows that the PDI value increased slightly as the storage time increased, and that it varied depending on the percentage of oleic acid. A smaller percentage of oleic acid resulted in a smaller PDI value. Oleic acid was selected for this study due to its biocompatible properties [31] and its potential to promote the stability of NEs [36]. As can been seen from the results in Table 3, all formulations had a PDI value of less than 0.7 over 14 days of storage, which indicates the formulations were stable. Sravanthi et al. [37] found that PDI values changed very little as the storage progressed, until week six of their study. In addition, the PDI value changed only minimally in a formulation of olive oil NE that was stored for three months at 4 °C and 25 °C [30]. Similarly, Ricaurte et al. [28] found that the PDI value increased as the percentage of oleic acid increased. 

Based on the RSM analysis, the particle size and PDI of the SBH NEs could be predicted using Equations (2) and (3), respectively. The coefficients of determination (*R*^2^) of all the prediction models were more than 0.90, indicating that only less than 10% of the total variation in the data obtained could not be explained by the model. The adjusted determination coefficient (*R*^2^*_adj_*) values for the models was more than 0.90, suggesting the models were reliable in predicting the experimental results. This showed that all the models obtained using RSM could be used to predict the particle size and PDI value of the SBH NEs. Yukuyama et al. [30] stated that the systematic design of their experiment was a success, that application of RSM optimisation delivered a novel range of critical processing parameters within the design space, and that similar experimental results could be achieved. Similar findings were obtained by Mehmood [21], who found that the predicted values agreed with the experimental results obtained through RSM design.

(2) $$\mathrm{Particle Size}=913.50-4.09A+197.46B-143.76C-143.54\mathrm{AB}+5.78\mathrm{AC}-119.33\mathrm{BC}+81.27A^{2}-753.38B^{2}+172.18C^{2}$$

(3) $$\mathrm{PDI}=+0.3719-0.0064A+0.0244B+0.0296C-0.0150\mathrm{AB}-0.0052\mathrm{AC}-0.0283\mathrm{BC}-0.0240A^{2}-0.1612B^{2}+0.0026C^{2}$$

The model revealed that the best formulation of SBH NEs was formulation A1, with 1% (*w*/*w*) oleic acid, kept at 4 °C. To verify the model obtained using RSM, an experimental validation was conducted and the results are tabulated in Table 5. Regarding the results for size and PDI, the percentage errors were very small, at less than 15%. The results show that formulation A1 was physically stable, as the experimental and predicted values of dependant variables were within the desired range. 

### 3.2. Characterisation of SBH and SBH NEs

#### 3.2.1. Fourier-Transform Infrared Spectroscopy (FTIR) Patterns of SBH and SBH NEs

**Figure 3:**
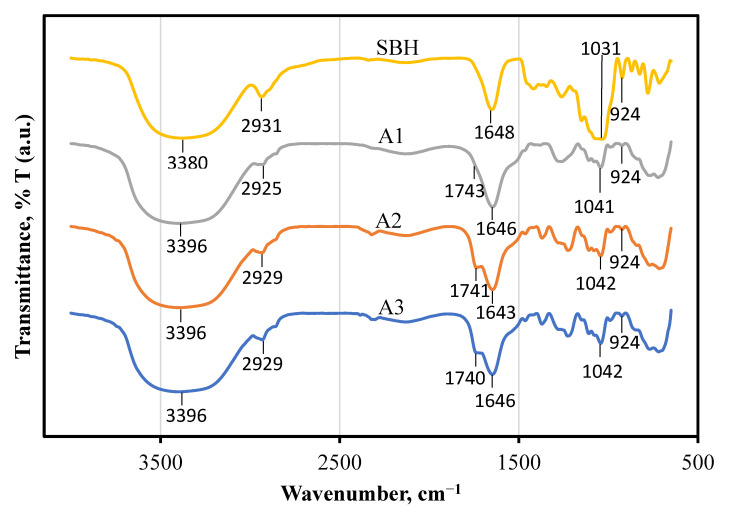
FTIR spectra of SBH and SBH NEs.

Figure 3 shows the variation of FTIR spectra 4000 to 650 cm^−1^ in formulations A1, A2, A3, and a control sample, SBH. The graphs clearly show spectral differences between functional groups. Dominant peaks shown in all FTIR (around 3396 cm^−1^ and 1648 cm^−1^) were attributed to OH stretching and OH deformation; the band around 2929 cm^−1^ was attributed to the C-H stretching in the carboxylic acids and NH_3_ stretching in the honey [38]. The band around 1648 cm^−1^ indicates the stretching of C=O in the carboxylic functional group, whereas the band at 1051 cm^−1^ and 1259 cm^−1^ corresponds to the stretching vibration of C–OH and C–O in the honey [39]. Next, the peak which appeared at 924 cm^−1^ was attributed to the stretching modes of carbohydrates in the honey, such as C-O and C-C [40]. However, observation of the formulations A1, A2, and A3 indicated a peak around band 1740 cm^−1^, which was attributed to the stretching vibration of C=O in the oleic acid [41]. Overall, the functional group of formulations A1, A2, A3 and the control sample, SBH, after being subjected to HPH for 5 cycles at 100 MPa at room temperature, was still the same as the functional group of the honey. Besides, it has been reported by Mail et al. [42] that the functional groups of SBH are almost identical to those of common honey produced by *Apis* bees. The authors also stated that SBH has lower absorption in carbohydrate regions and high absorption in water regions due to its natural chemical constituents. 

#### 3.2.2. Investigation of the SBH NEs’ Morphology Using Transmission Electron Microscopy

**Figure 4:**
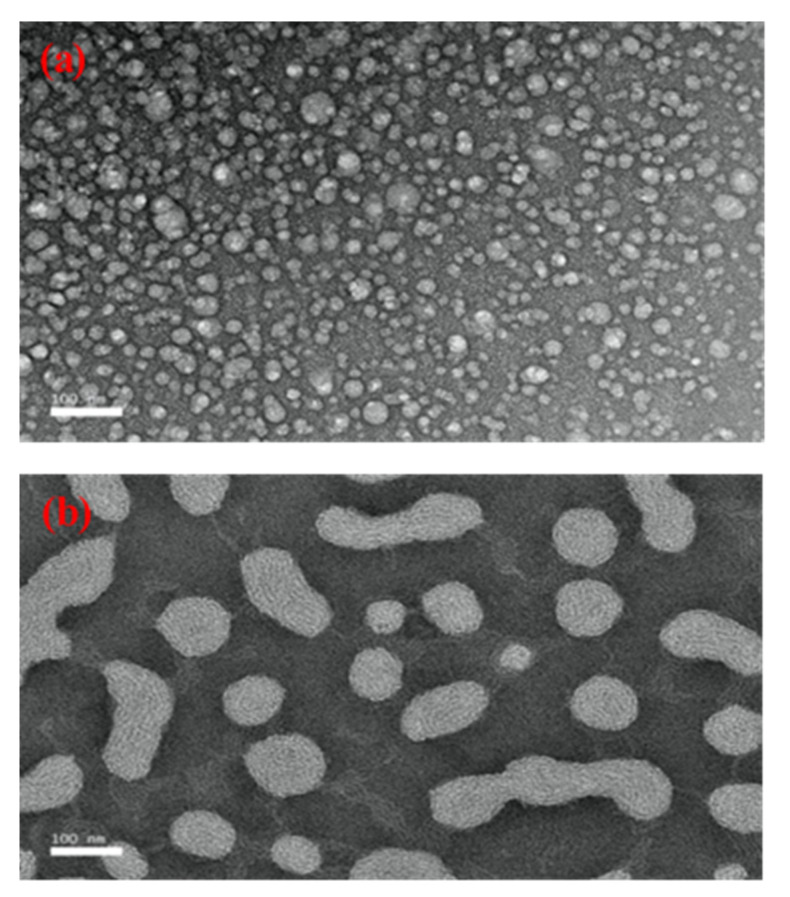
TEM images of SBH NEs without oleic acid (**a**), and with 1% (*w*/*w*) oleic acid (**b**).

TEM images were used to confirm the particle size of formulation A1 and to compare its morphology with the control sample, SBH. As shown in Figure 4a, the particle size of SBH was less than 100 nm. As shown in Figure 4b, the particle size of formulation A1 with 1% (*w*/*w*) oleic acid was also less than 100 nm, but larger than the control sample. Though formulation A1 matched the result obtained by the particle size analyser, the differences from the particle size of SBH were large. The difference in particle size between A1 and SBH may be due to the presence of oleic acid. Furthermore, the results for the SBH sample showed a spherical-shaped particle, similar that observed by Prakash et al. [43] and Lu et al. [44], who studied linalool NEs and citral NEs, respectively. These authors reported that the observed NEs were spherical in shape and their size matched the results from the particle-size analyser. However, for A1, some of the droplets shown were an irregular shape. This may be due to the presence of oleic acid, which has a different viscosity than SBH. Abdou et al. [45] also found a similar result for droplets in curcumin-sunflower oil NEs, which were not spherical in shape due to the difference in viscosity of the materials used. As shown in Figure 4b, the droplets of formulation A1 were not evenly distributed due to the presence of some irregular-shaped droplets. This contradicts the findings of Saari et al. [46], who found an even distribution and spherical-shaped droplets in their study of curcumin-loaded coconut oil and honey NEs. 

## 4. Conclusions

HPH was used to produce SBH NEs according to three different formulations: A1, A2, and A3. The SBH NEs were characterized and evaluated with regard to particle size, stability, and morphology. The SBH NEs were optimised using RSM, and this process indicated that formulation A1 was the best of the three, with R^2^ values of more than 0.90. The findings were consistent and proven by an experimental validation of less than 10% error. The experiment also demonstrated that formulation A1 was physically stable, with values for particle size and PDI within the desired range. The results from TEM showed that the particle size of the formulation A1 was less than 100 nm. However, the shapes of the particles in formulation A1 were irregular, which makes the distribution uneven, as shown in the TEM images. This irregularity in shape could be due to the difference in viscosity of SBH and oleic acid. The results of this study show that SBH NEs can be developed using HPH and have the potential to be used in many industries, including pharmaceuticals, food, and cosmetics.

## Figures and Tables

**Table 1:** Formulation of NEs.

Materials (*w*/*w* %)	Formulation		
	A1	A2	A3
Oleic acid	1	3	5
Glycerol	8	8	8
Tween 80	5	5	5
Distilled water	71	69	67
SBH	15	15	15

**Table 2:** Three-level factorial design experiment factors and levels.

Code	Independent Variables	Level		
		−1	0	1
A	Temperature (°C)	4	28	40
B	Oleic acid (*w*/*w* %)	1	3	5
C	Storage time (weeks)	1	2	3

**Table 3:** Three-level factorial response surface design arrangement and response for SBH NEs.

Run	Independent Variables			Response Variables	
	Temperature	Oleic Acid	Storage Time	PDI	Size
1	−1	−1	1	0.21	39.13
2	−1	−1	0	0.15	36.49
3	0	−1	1	0.22	81.73
4	1	−1	1	0.25	411.70
5	1	−1	−1	0.10	256.14
6	−1	1	0	0.22	460.10
7	0	0	−1	0.34	849.26
8	1	0	0	0.32	592.17
9	1	0	−1	0.34	1031.00
10	0	0	0	0.38	570.70
11	−1	0	1	0.39	551.76
12	1	1	0	0.16	245.80
13	1	1	−1	0.22	518.86
14	0	−1	−1	0.10	35.35
15	1	0	1	0.36	609.83
16	0	0	1	0.39	499.43
17	−1	−1	−1	0.10	35.35
18	1	1	1	0.20	195.30
19	0	−1	0	0.24	51.43
20	0	1	−1	0.22	518.86
21	0	1	0	0.25	294.76
22	−1	1	−1	0.22	709.22
23	−1	1	1	0.27	413.03
24	0	1	1	0.20	263.76
25	−1	0	−1	0.34	1031.00
26	−1	0	0	0.33	608.43
27	1	−1	0	0.16	176.76

**Table 4:** Analysis of variance for the particle size and PDI of SBH NEs’ response surface quadratic model.

Particle Size					
Source	Sum of Square	Degree of Freedom	Mean Aquare	*F*-Value	*p*-Value
Model	5.006 × 10^6^	9	5.562 × 10^5^	28.42	<0.0001
A—Temperature	300.78	1	300.78	0.015	0.9028
B—Oleic acid	6.895 × 10^5^	1	6.895 × 10^5^	35.24	<0.0001
C—Storage time	3.655 × 10^5^	1	3.655 × 10^5^	18.68	0.0005
Residual	3.327 × 10^5^	17	19,568.57		
Corrected total	5.338 × 10^6^	26			
**PDI**					
**Source**	**Sum of Square**	**Degree of Freedom**	**Mean Square**	***F-*** **Value**	***p*** **-Value**
Model	0.1962	9	0.0218	31.17	<0.0001
A—Temperature	0.0007	1	0.0007	1.07	0.3157
B—Oleic acid	0.0106	1	0.0106	15.11	0.0012
C—Storage time	0.0155	1	0.0155	22.20	0.0002
Residual	0.0119	17	0.0007		
Corrected total	0.2081	26			

**Table 5:** Experimental validation.

Optimum Condition	Coded Level	Actual Level
Oleic acid (*w/w* %)	−1	1
Storage time (days)	0	14
**Results**	**Particle Size (nm)**	**PDI**
Predicted	46.43	0.219
Actual	49.56	0.201
Error (%)	6.74	8.22
